# Symptomatic unilateral idiopathic giant bullous emphysema : a case report

**DOI:** 10.1186/s12890-022-02135-3

**Published:** 2022-09-09

**Authors:** S. Garvey, J. Faul, L. Cormican, D. Eaton, E. P. Judge

**Affiliations:** 1 Department of Respiratory Medicine, Connolly Hospital, Blanchardstown, Dublin 15, Ireland; 2 Department of Cardiothoracic Surgery, Mater Misericordiae University Hospital, Eccles St, Dublin 7, Ireland

**Keywords:** Giant bullous emphysema, Solitary bulla, Vanishing lung syndrome, Bullectomy, Case report

## Abstract

**Background:**

Idiopathic Giant Bullous Emphysema (or Vanishing Lung Syndrome) is a rare condition which is usually associated with male gender, active smoking and underlying emphysematous disease. We present an unusual case of a giant bulla occurring in the absence of these risk factors.

**Case presentation:**

A 54-year-old woman presented to the respiratory outpatient clinic with gradually worsening left sided chest discomfort, which was most marked during a recent flight. She had no significant dyspnoea or other symptoms. She had a remote 5-pack-year smoking history. Chest X-Ray revealed a large hyperlucent area in the left upper lobe. CT Thorax found this to be an isolated bulla occupying more than one-third of the hemithorax. The remaining lung parenchyma was normal. A diagnosis of Idiopathic Giant Bullous Emphysema was made. The patient was referred for VATS (Video-assisted thoracoscopic surgery) bullectomy which was carried out without complication. Her symptoms resolved completely following the operation.

**Conclusions:**

This is an unusual case of a solitary giant bulla occurring without major risk factors or underlying lung disease. VATS bullectomy was shown to be an effective therapeutic option, allowing re-expansion of compressed lung tissue and complete resolution of symptoms.

## Background

Idiopathic Giant Bullous Emphysema (iGBE), or Vanishing Lung Syndrome (VLS), is a rare condition first described in 1937 [1]. It is defined by the presence of large bullae occupying at least one-third of a hemithorax and causing compression of the surrounding lung parenchyma [2]. This is a condition which classically occurs in males with heavy smoking histories and is associated with underlying emphysema.

## Case presentation

A 54-year-old woman presented with intermittent left-sided chest discomfort over 2 years. She described this symptom as a “pressure” sensation in the anterior chest which was brought on by physical strain such as heavy lifting. She also reported that this discomfort was more noticeable and severe during air travel over recent years, with discomfort peaking during the middle of the flight and easing with descent. The most recent flight-related.

Episode was particularly severe and this is what prompted her presentation. She had no dyspnoea or other symptoms of note.

On further discussion, she revealed that she had been followed-up in the respiratory outpatient clinic of a different hospital some 10 years previous for an incidentally noted large bulla in her left lung. Following 5 years of annual surveillance imaging, she was discharged as she had remained asymptomatic and the bulla had not increased in size.

She was a smoker in her youth, accumulating a 5-pack-year history and quitting before the age of 30. She had no medical history and no exposures of note. There was no family history of pneumothorax, lung or connective tissue disease.

Examination revealed no hypoxia, digital clubbing or peripheral stigmata of respiratory disease. Auscultation revealed equal bilateral air entry with normal breath sounds.

**Fig. 1:**
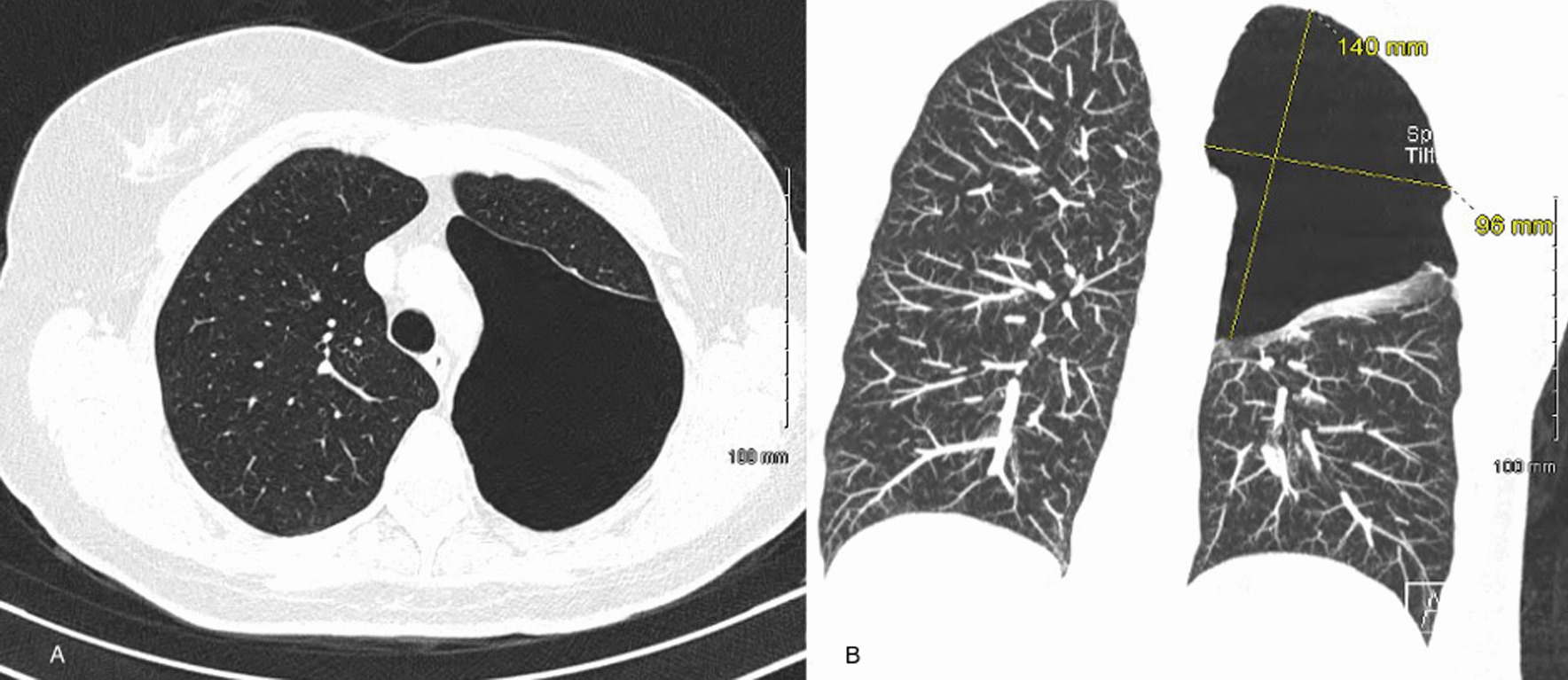
Initial CT imaging; **a** axial view, **b** coronal view. CT Imaging demonstrating a solitary giant bulla in the left upper lobe with normal surrounding parenchyma and secondary compression of the left upper and lower lobes

A chest X-ray was done which revealed a large bulla in the left upper zone. This was followed by a high-resolution CT of the thorax (Fig. 1) which demonstrated a large avascular, air-filled region occupying the upper half of the left hemithorax, consistent with a large bulla. A diagnosis of iGBE was made. There was compression of surrounding lung. There was no evidence of emphysematous lung disease (paraseptal or centrilobular) in the remainder of both lungs.

Pulmonary function tests showed normal spirometry and diffusion capacity. Lung volumes were not performed. A connective tissue disease blood panel and *α*1 anti-trypsin level were unremarkable.

**Fig. 2:**
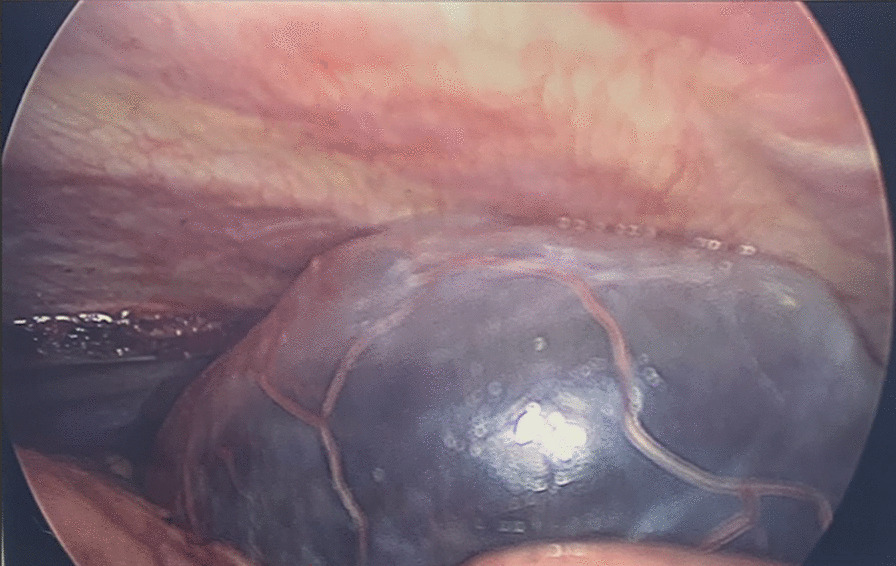
Intra-operative image of the giant bulla that was resected during VATS Bullectomy

**Fig. 3:**
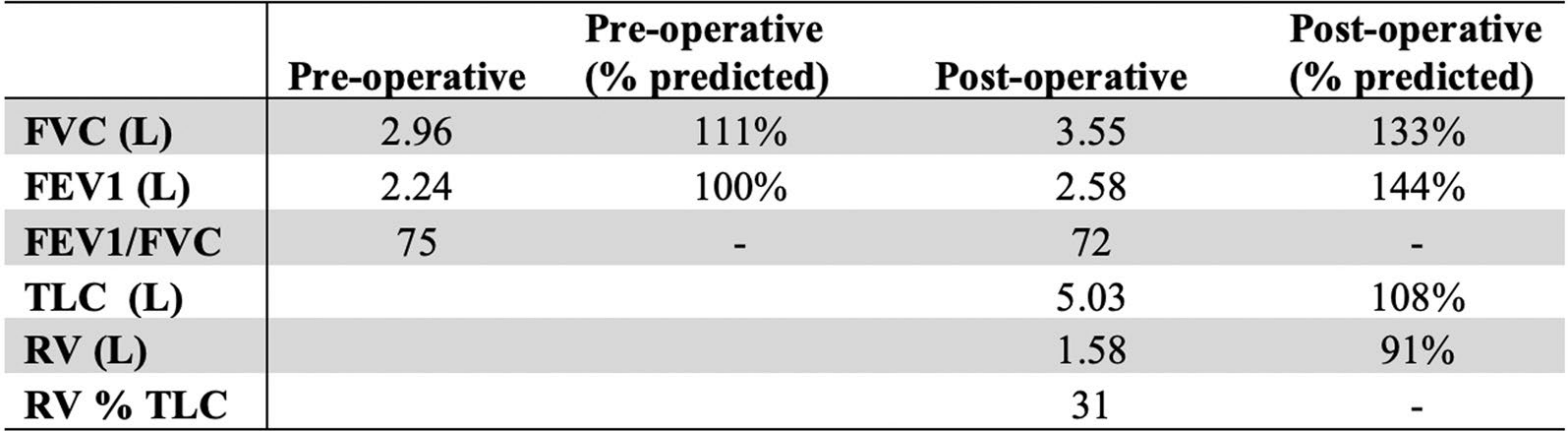
Pulmonary Function Tests. Spirometry demonstrating improvement in forced vital capacity (FVC) and forced expiratory volume in 1st second (FEV1) post-operatively. Lung volumes including total lung capacity (TLC) and residual volume (RV) were not performed pre-operatively. Attempts to measure diffusion capacity were limited by patient technique on both occasions

She was referred for surgical assessment and a VATS Bullectomy was performed (Fig. 2). This was a two-port procedure. The bulla was intentionally ruptured, creating space in the hemithorax. Adhesions within the surrounding structures were divided. The bulla was resected with a small rim of normal tissue. A 24 french drain was sited and the patient was then extubated. She tolerated this procedure well and without complication. At her recent 6-month follow-up, she was doing well and her previously described symptoms had completely resolved. Repeat spirometry is significantly improved and lung volumes are within normal limits (Fig. 3).

## Discussion and conclusions

Previous case reports and case series have described the clinical characteristics of iGBE [2, 3]. Patients are typically male, and there is a strong association with tobacco smoking and chronic obstructive pulmonary disease (COPD). Of the 18 patients described between two case series, there was just one female and one non-smoker. Another case series described five cases of iGBE within three generations of a single family, none of whom were smokers [4]. This highlights the possibility of a role for genetic susceptibility in the pathogenesis of this condition. In this case, we present a female patient who had a remote, minimal smoking history, no evidence of COPD and no family history of note.

iGBE usually presents either asymptomatically as an incidental finding, or with dyspnoea which can usually be correlated with airflow obstruction on PFTs. In contrast, this case of iGBE actually presented with chest discomfort, which was associated with straining and exacerbated at high altitude. This presentation likely reflects the effect of alterations in thoracic pressure on the volume of the bulla [5]. This can be explained by Boyle’s Law which describes the inverse relationship between pressure and volume of gases. Atmospheric pressure at ground level is 760 mmHg and this decreases with increasing altitude. At cruising altitude, it is possible that a bulla could expand by up to 35% in the context of a cabin pressure of about 560mmHg [6]_._ This significant expansion could cause compression of the surrounding lung and mediastinum, leading to chest pain. A small handful of reports have described giant bullae which have come to medical attention only following symptoms which have developed exclusively at altitude. Symptoms have ranged from chest pain and breathlessness to loss of consciousness with transient neurological findings [7].

Radiologically, iGBE typically causes bilateral, asymmetric, paraseptal bullous disease. All of the 18 patients in the two previously-mentioned case series had multiple large bullae on imaging [2, 3]. There are very few reported cases of iGBE presenting as unilateral solitary bullae. One such case reported an isolated giant bulla in the right middle lobe of a non-smoking female patient [8]. In contrast, our report the describes a large bulla occurring in the left upper lobe.

Giant bullectomy is indicated for symptomatic patients. Resection of giant bullae allows the compressed lung to re-expand, thereby improving V/Q matching and decreasing both dead space and residual volume [9]. Patient selection is important to minimise risk and maximise benefit. The best results are obtained in patients with large bullae and in whom compression of a significant volume of potentially functional surrounding lung parenchyma can be demonstrated [10]. This case highlights how bullectomy was an effective therapeutic approach that resulted in full resolution of the patient’s symptoms.

## Data Availability

All data sourced was obtained directly from the patient’s confidential medical file. No dataset was generated or analysed in writing this case report.

## Abbreviations

iGBE;: Idiopathic giant bullous emphysema
VLS;: Vanishing lung syndrome
VATS;: Video-assisted thoracoscopic surgery
COPD;: Chronic obstructive pulmonary disease
